# The Abnormal Functional Connectivity in the Locus Coeruleus-Norepinephrine System Associated With Anxiety Symptom in Chronic Insomnia Disorder

**DOI:** 10.3389/fnins.2021.678465

**Published:** 2021-05-21

**Authors:** Liang Gong, Min Shi, Jian Wang, Ronghua Xu, Siyi Yu, Duan Liu, Xin Ding, Bei Zhang, Xingping Zhang, Chunhua Xi

**Affiliations:** ^1^Department of Neurology, Chengdu Second People’s Hospital, Chengdu, Sichuan, China; ^2^Department of Neurology, The Third Affiliated Hospital of Anhui Medical University, Heifei, China; ^3^Department of Acupuncture and Tuina, Chengdu University of Traditional Chinese Medicine, Chengdu, China; ^4^Department of General Practice, Chengdu Second People’s Hospital, Chengdu, China

**Keywords:** insomnia, locus ceruleus-norepinephrine, anxiety, functional connectivity, anterior cingulate cortex

## Abstract

**Background:**

Mental syndromes such as anxiety and depression are common comorbidities in patients with chronic insomnia disorder (CID). The locus coeruleus noradrenergic (LC-NE) system is considered to be crucial for modulation of emotion and sleep/wake cycle. LC-NE system is also a critical mediator of the stress-induced anxiety. However, whether the LC-NE system contributes to the underlying mechanism linking insomnia and these comorbidities remain unclear. This study aimed to investigate the LC-NE system alterations in patients with insomnia and its relationship with depression and anxiety symptoms.

**Materials and Methods:**

Seventy patients with CID and 63 matched good sleep control (GSC) subjects were recruited and underwent resting-state functional MRI scan. LC-NE functional network was constructed by using seed-based functional connectivity (FC) analysis. The alterations in LC-NE FC network in patients with CID and their clinical significance was explored.

**Results:**

Compared with GSC group, the CID group showed decreased left LC-NE FC in the left inferior frontal gyrus, while they had increased LC-NE FC in the left supramarginal gyrus and the left middle occipital gyrus (MOG). For the right LC-NE FC network, decreased FC was found in left dorsal anterior cingulate cortex (dACC). Interesting, the increased LC-NE FC was located in sensory cortex, while decreased LC-NE FC was located in frontal control cortex. In addition, the FC between the left LC and left MOG was associated with the duration of the disease, while abnormal FC between right LC and left dACC was associated with the anxiety scores in patients with CID.

**Conclusion:**

The present study found abnormal LC-NE functional network in patients with CID, and the altered LC-NE function in dACC was associated with anxiety symptoms in CID. The present study substantially extended our understanding of the neuropathological basis of CID and provided the potential treatment target for CID patients who also had anxiety.

## Introduction

Insomnia is a prevalent and limiting condition affecting daytime functioning. The chronic insomnia disorder (CID) affects an estimated 10% of the population; in particular during the global lockdown due to the COVID-19 outbreak, insomnia prevalence has doubled to tripled (Kokou-Kpolou et al., 2020; Shi et al., 2020). Insomnia is also highly comorbid with other mental health disorders, especially depression and anxiety (Blake et al., 2018). However, the neuropathological basis of these comorbidities is still unclear.

Numerous groups have used various neuroimaging approaches and demonstrated specific brain structural and functional alterations in patients with CID (Huang et al., 2012; Joo et al., 2013; Li et al., 2018; Yu et al.,2018a,b; Gong et al., 2020c). However, these studies have yielded diverse findings, e.g., the altered brain structural regions included orbitofrontal cortex, hippocampus, amygdala, precuneus, and anterior cingulate cortex (ACC) (Altena et al., 2010; Winkelman et al., 2013), while the abnormal functional networks included default mode network, salience network, frontostriatal network, and reward networks (Chen et al., 2014; Huang et al., 2017). Recently, Tahmasian et al. (2018) performed activation likelihood estimation to identify consistent patterns of abnormal brain alterations in insomnia disorder and found no significant convergent evidence across previous studies. The diverse finding of brain alterations in patients with insomnia could be possibly explained by the heterogeneous neuroimaging approaches (Liu et al., 2018). In addition, the heterogeneous clinical features of insomnia should be considered, especially the high comorbidity symptoms like depression and anxiety (Sivertsen et al., 2012; Blake et al., 2018; Bragantini et al., 2019; Gong et al.,2020a,b; Sweetman et al., 2020). Thus, investigation of the brain mechanism underlying CID and mental symptom comorbidities is essential for comprehensive understanding of insomnia.

The locus coeruleus (LC) is a pontine nucleus belonging to the ascending reticular activating system (Moruzzi and Magoun, 1949), which produces the majority of brain norepinephrine (NE). The locus coeruleus noradrenergic (LC-NE) system is considered to be the crucial system involved in the regulation of physiological functions, including waking, arousal and attention (Ambrosini et al., 2013; Szabadi, 2013; Lovett-Barron et al., 2017). Previous studies indicated that excessive activity in the LC-NE system was incompatible with sleep and may contribute to insomnia (Berridge et al., 2012). The 24-h plasma NE level was lower in insomnia patients than in good sleepers, and the decreased NE level was associated with poorer sleep quality in the insomnia group (Grimaldi et al., 2020). Hyperarousal and sleep reactivity are the key components in the pathophysiology model of CID (Kalmbach et al., 2018). In addition, LC-NE system is a critical mediator of the stress-induced anxiety and a potential intervention target for stress-related depressive and anxiety disorders (McCall et al., 2015). Previous neuroimaging studies have indicated that the resting-state functional connectivity (FC) analysis of LC was feasible to detect LC-NE function in human brain (Zhang et al., 2016; Song et al., 2017). Recently, using LC FC analysis approach, the disrupted LC FC have been found in healthy adults with parental history of Alzheimer’s disease (Del Cerro et al., 2020b) and patients with late-life depressive disorder (Del Cerro et al., 2020a). However, whether the LC-NE system contributes to the underlying mechanisms linking insomnia and these comorbidities remains unclear.

We aimed to investigate the LC-NE system alteration in patients with insomnia and its relationship with the depression and anxiety symptoms. To this end, we analyzed the LC-NE FC network in a group of patients with CID using resting-state functional MRI (rs-fMRI) data. Due to the role of LC in the regulation of behavioral state, we hypothesized that both increased and decreased brain regions could be found in the LC-NE network. Furthermore, we hypothesized that the altered LC-NE system would be associated with mental symptoms in patients with CID.

## Materials and Methods

### Participants

Seventy patients with CID and 63 matched good sleep control (GSC) subjects were recruited in the present study. The study was approved by the local ethical committee (the Institutional Review Board of the third affiliated hospital of Anhui Medical University). All participants were of Chinese Han descent and right handed, and written informed consent was provided. Two patients with CID and three healthy participants were excluded due to excessive head motion artifacts (exceeding 1.5 mm or 1.5° of angular motion relative to the first volume). Therefore, 68 patients with CID and 60 GSCs were included in the final analysis.

For inclusion in the CID group, patients were required to meet the diagnostic criteria for CID outlined in the International Classification of Sleep Disorders, third version (Sateia, 2014). Additional inclusion criteria for the CID group were as follows: (1) had not taken any hypnotic and antidepressant medication 2 weeks before neuropsychological test and MRI scan; and (2) aged 18–55 years with age at onset under 50 years. The exclusion criteria were: (1) a history of other neuropsychiatric disorders; (2) a history of substance abuse (caffeine, nicotine, and alcohol); and (3) contraindications to MRI. GSC subjects were required to meet the following criteria: (1) good sleep and mood and normal cognitive function; (2) no history of neurological and psychiatric disease, seizures, head injury, stroke, or transient ischemic attack; (3) no caffeine, drug, or alcohol abuse; and (4) no brain lesions found by a regular T2-weighted MRI scan.

### Behavioral Assessments

Pittsburgh Sleep Quality Index (PSQI) was used to assess the subjective sleep quality in CID group (Buysse et al., 1989; Backhaus et al., 2002) and to measure the insomnia severity. The self-rating depression scale (SDS) was used for the depression evaluation, while the Zung’s self-rating anxiety scale (SAS) was used for the anxiety evaluation (Zung et al., 1965; Zung, 1971).

### Imaging Acquisition and Preprocessing

Imaging was performed at the Third Affiliated Hospital of Anhui Medical University using a Siemens Verio 3.0-Tesla scanner (Siemens, Erlangen, Germany). MRI data acquisition was carried out between 4 p.m. and 6 p.m. in all participants. Structural images were acquired using a high-resolution spoiled gradient-recalled echo sequence with the following parameters: repetition time/echo time (TR/TE) = 1,900/2.48 ms; flip angle (FA) = 9°; acquisition matrix = 256 × 256; field-of-view = 240 × 240 mm; thickness = 1.0 mm; gap = 0 mm; number of slices = 176; number of excitations = 1.0. The rs-fMRI datasets were obtained using an 8-min gradient-recalled echo-planar imaging pulse sequence with the following parameters: TR/TE = 2,000/35 ms; FA = 90°; acquisition matrix = 64 × 64; thickness = 3.5 mm; number of slices = 36. Other parameters were the same as those utilized for structural images. During scanning, all participants were instructed to relax and keep their eyes closed, and stabilizers were used to immobilize the head. Wakefulness was assessed following scanning, and all participants claimed to be awake during the study.

The rs-fMRI data were preprocessed using SPM12^1^ and the DPABI 4.3 (Data Processing & Analysis of Brain Imaging^2^) implemented in MATLAB 8.0 (The MathWorks, Inc., Natick, MA, United States) (Yan et al., 2016). We removed the first five initial volumes to account for the magnetization equilibrium and adaptation to the experimental environment. The remaining 235 images were then slice-time corrected, reoriented, realigned, and coregistered to the T1-weighted structural images, which were segmented using DARTEL (Ashburner, 2007). The participants with excessive head motion above 1.5 mm or 1.5° of angular motion relative to the first volume were removed for next analyses. There was no significant difference in mean framewise displace between groups (CID group: 0.071 ± 0.045, GSC group: 0.082 ± 0.061; *p* = 0.42). The correlation between FD and behavioral information were calculated in the CID group. Images from all participants were normalized into standard stereotactic Montreal Neurological Institute space and smoothed using a Gaussian kernel (full width at half-maximum = 6 mm). Voxel time series were further detrended and temporally filtered (0.01–0.1 Hz). We normalized the variance of each time series to control for fluctuations in signal intensity. Noise associated with white matter/cerebrospinal fluid signals and 24 head motion-related covariates were regressed out. The global signal was not regressed out given the controversy regarding its application to rs-fMRI data (Murphy et al., 2009; Chai et al., 2012).

### LC-NE Functional Network Construction

Bilateral, LC-based, voxel-wise FC analysis was conducted using the DPABI 4.3. Bilateral LC was selected as the seed region to construct the LC-NE functional network, separately. To avoid the localization error of LC by “in-house” approach, the bilateral LC seed regions were extracted from the automated anatomical labeling atlas 3 (AAL3) (Rolls et al., 2020). The average time course in each LC region, defined as the seed time course, was correlated with the time course in all brain voxels using Pearson’s cross correlation. A Fisher’s Z-transformation was applied to improve the correlation coefficients (CC) so that they approached normal distribution [*Z* = 0.5ln(1 + CC)/(1 − CC)] (Lowe et al., 1998; Liu et al., 2017). Thus, a map of each individual pattern in LC FC network was separately obtained.

### Statistical Analysis

First, two independent *t* tests and chi-square tests (only for the gender) were performed for the demographic and behavior data comparison between the two groups (SPSS 24.0; SPSSInc., Chicago, Ill). Pearson correlation analysis was employed to detect the clinical features (PSQI, SDS, SAS, and duration of disease) associations in the CID group. Statistical significance was set at *p* values < 0.05.

Second, a voxel-wise, two independent *t* test was used to obtain the group difference of LC-NE networks after removal of the effects of covariates including gender and age. The voxel-level significance threshold was set at a *p* value < 0.001, corrected for multiple comparisons at the cluster level using the 3dClustSim program in AFNI_18.3.03 (gray matter mask correction: 67,541 voxels, estimated smoothness is 14.53 mm of statistic image, voxel-level *p* value < 0.001, cluster level α value < 0.001, κ > 35, cluster size > 945 mm^3^)^3^. The peak *Z* value in the region of interest was reported by xjview^4^. As the gender difference of LC-NE FC network was reported in a previous study (Zhang et al., 2016), we also explore the gender difference of LC-NE network in patients with CID.

Third, to further explore the clinical significance of the altered LC-NE FC in CID patients, a partial correlation analysis was used to investigate the association between the altered LC-NE and PSQI, SAS, and SDS scores, controlling for the effects of age, gender, and disease duration. Significance was set at a *p* value < 0.05 after correction for multiple comparisons using false-discovery rate (FDR) correction.

## Results

### Demographic and Behavioral Information

Demographic data and behavior performance in two groups are shown in Table 1. No significant differences in gender, age, and education were found between GSC subjects and CID patients. The mean score of PSQI (severity of insomnia) in CID group was 14.6, ranging from 11 to 19; the mean duration of insomnia was 4.45 years, ranging from 0.25 to 16.67 years. The mean depression and anxiety scores were 52.35 (range: 18–68) and 53.88 (range: 36–65), respectively. According to cut-off value of Chinese version of SDS and SAS scales (53 for SDS and 50 for anxiety), 23 CID patients presented depressive symptom and 49 CID patients displayed anxiety symptoms. The Pearson correlation analyses revealed that the SAS was positively associated with PSQI scores in the CID group (*r* = 0.344, *p* = 0.005). No other significant correlation was found between clinical features. No significant correlation was found between FD and clinical features (all *p* > 0.05).

**TABLE 1:** Demographic and clinical traits for all participants.

Characteristic	CID (*n* = 68)	GSC (*n* = 60)	*T/X*^2^	*p* value
				
Age	39.85 ± 11.18	39.47 ± 10.40	0.20	0.43
Gender (M/F)	32/36	25/35	0.37	0.54
Education (years)	11.26 ± 2.77	11.90 ± 4.20	1.02	0.31
PSQI	14.06 ± 1.96	–	–	–
SDS	52.35 ± 7.65	–	–	–
SAS	53.88 ± 5.00	–	–	–
Duration (Years)	4.45 ± 0.4.50	–	–	–

*CID, chronic insomnia disorder; GSC, good sleep controls; PSQI, Pittsburgh sleep quality index; SDS, Zung self-depression scale; SAS, Zung self-anxiety scale.*

### Altered LC-NE Functional Network in CID

**FIGURE 1:**
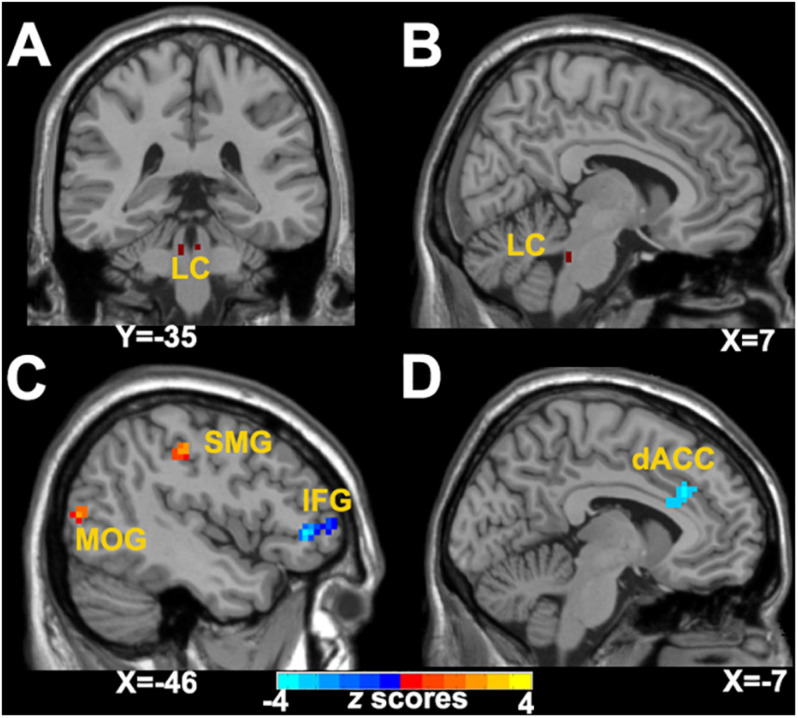
Abnormal LC-NE functional network in patients with CID. **(A**,**B)** LC seed selected from AAL 3 atlas. **(C)** Altered brain regions in the left LC-NE functional network in patients with CID are found in the left IFG, IPL, and MOG. **(D)** Altered brain regions in the right LC-NE functional network in patients with CID are found in the left dACC. LC-NE, locus coeruleus-norepinephrine; CID, chronic insomnia disorder; IFG, inferior frontal gyrus; MOG, middle occipital gyrus; SMG, supramarginal gyrus; dACC, dorsal anterior cingulate cortex; AAL, automated anatomical atlas.

As illustrated in Table 2 and Figure 1 for the left LC-NE FC network, compared with the GSC group, CID patients showed decreased FC in the left inferior frontal gyrus (IFG), while they had increased FC in the left supramarginal gyrus (SMG) and left middle occipital gyrus (MOG). For the right LC-NE FC network, CID patients had decreased FC in the dorsal anterior cingulate cortex (dACC). Taken together, the altered regions in the LC-NE network were all located in the efferent pathway, and the areas with increased FC were located in the sensory cortex, while those with decreased FC were found in the prefrontal cortex.

**TABLE 2:** Group differences in bilateral LC-NE FC network.

Brain region	BA	Voxel size	MNI coordinates (RAI)			Peak *Z* score
			*x*	*y*	*Z*	
**Left LC-NE FC network**						
Left IFG	47	50	−45	39	0	–4.33
Left MOG	19	202	−42	−81	9	4.65
Left SMG	40	56	−39	−30	39	4.46
**Right LC-NE FC network**						
Left dACC	24	57	−3	24	18	–3.86

*LC-NE FC, locus coeruleus-norepinephrine functional connectivity; BA, Brodmann’s area; MNI, Montreal Neurologic Institute; RAI, the right anterior and inferior direction is positive value; IFG, inferior frontal gyrus; MOG, middle occipital gyrus; SMG, supramarginal gyrus; dACC, dorsal anterior cingulate cortex.*

### Gender Difference of the Altered LC-NE Functional Network in the CID Group

**FIGURE 2:**
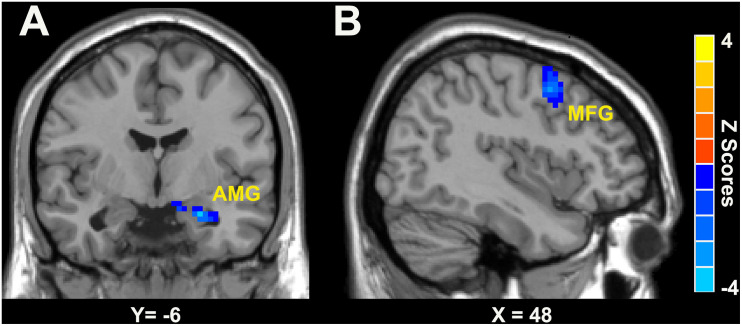
Gender differences of LC-NE functional network in patients with CID was located in AMG **(A)** and MFG **(B)**. The blue color bar means the LC-NE FC in female is lower than that in male. AMG, amygdala; MFG, middle frontal gyrus.

As shown in Figure 2 and Table 3, in the right LC-NE functional network, the LC-NE FC in the left amygdala and the right middle frontal gyrus (MFG) was greater in male when compared with female in the CID group. No significant gender difference was found in the left LC-NE functional network.

**TABLE 3:** Gender differences in bilateral LC-NE FC network in CID group.

Brain region	BA	Voxel size	MNI coordinates (RAI)			Peak *Z* score
			*X*	*y*	*z*	
**Left LC-NE FC network**						
None						
**Right LC-NE FC network**						
Left AMG	36	59	−24	−6	−21	−4.51
Right MFG	6	112	48	12	48	−3.95

*LC-NE FC, locus coeruleus-norepinephrine functional connectivity; BA, Brodmann’s area; MNI, Montreal Neurologic Institute; RAI, the right anterior and inferior direction is positive value; AMG, amygdala; MFG, middle frontal gyrus.*

### Clinical Significance of the Altered LC-NE Functional Network

**FIGURE 3:**
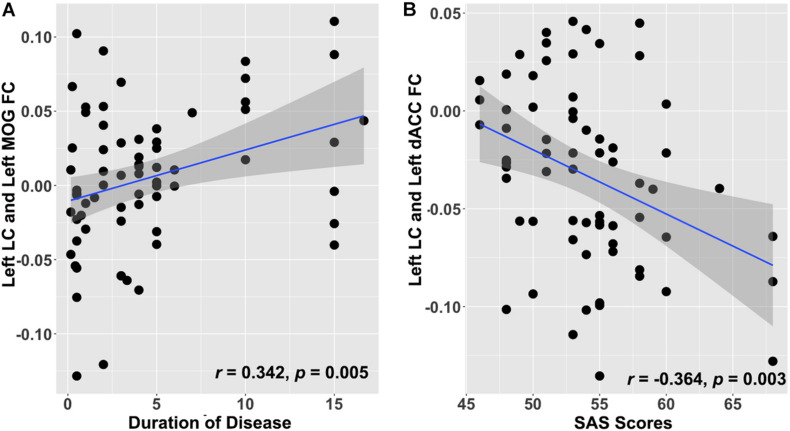
Clinical significance of altered LC-NE functional network in patients with CID. **(A)** Increased LC-NE FC in left MOG is positively associated with duration of insomnia. **(B)** Decreased LC-NE FC in the left dACC is negatively associated with anxiety symptom in patients with CID. LC-NE, locus coeruleus-norepinephrine; CID, chronic insomnia disorder; MOG, middle occipital gyrus; dACC, dorsal anterior cingulate cortex.

A partial correlation analysis was conducted to investigate the relationship between abnormal LC-NE FC and clinical features in patients with CID. As shown in Figure 3, after FDR correction, FC between the left LC and left MOG was positively correlated with the duration of disease in patients with CID, after controlling for the effects of gender and age (*r* = 0.342, *p* = 0.005, *p*FDR = 0.042). In addition, FC between right LC and left dACC was negatively correlated with the SAS scores in patients with CID, after controlling for the effects of gender and age (*r* = −0.364, *p* = 0.003, *p*FDR = 0.036). No other significant correlation was found after FDR correction (*p*FDR > 0.05 in all cases).

## Discussion

Our study demonstrated that patients with CID presented both increased and decreased regions in LC-NE functional network when compared with GSC subjects. The areas with increased FC were located in the posterior sensory cortex of the LC-NE functional network, while those with decreased FC were found in the prefrontal cortex of the LC-NE functional network. Importantly, the altered LC-NE function in dACC was associated with anxiety symptoms in patients with CID. These findings indicated that there was a dysfunctional LC-NE system in patients with CID, and that this abnormal LC-NE functional network would contribute to the combined anxiety symptoms in these patients. The present study substantially extended our understanding of the neuropathological basis of CID and provided the potential treatment target for CID patients who had anxiety.

As expected, areas with both increased and decreased LC-NE FC were found in patients with CID. Anatomically, the ascending pathway of LC-NE system is projected to the limbic system, thalamus, basal forebrain, and the entire neocortex (Szabadi, 2013); the areas with abnormal LC-NE FC network in patients with CID were found in the neocortex. We found that in patients with CID, areas with increased LC-NE FC were located in SMG and MOG. The SMG is part of somatosensory association cortex, which is involved in tactile sensory, and space and limb location perception (Leichnetz, 2011), whereas the MOG is involved in visual and spatial information processing (Renier et al., 2010). Interestingly, the areas with increased LC-NE FC were located in the posterior sensory cortex. These findings could elucidate that CID patients suffer from the hypersensitivity in sensory perception of tactile, visual, and auditory stimuli. The present findings supported the notion of the hyperarousal state of the posterior sensory cortex in the LC-NE ascending pathway in patients with CID.

Areas with decreased LC-NE FC were found in the left dACC and IFG in patients with CID. The dACC is the important region for decision making in executive control network (Bush et al., 2002) and is also involved in behavioral adaptation (Logiaco et al., 2015). The decreased LC-NE network might indicate decreased self-control and adaptation of the arousal in the CID patients. The IFG is also an important region in prefrontal cortex and a core node in executive control network. Recent neuroimaging studies also found lower degree centrality, amplitude of low-frequency fluctuations, and functional connectivity between IFG and orbital frontal cortex in the IFG in patients with insomnia than in healthy controls (Li et al., 2016; Yan et al., 2018; Xie et al., 2020). In addition, the FC in IFG was associated with the duration of the disease, anxiety, and insomnia symptoms in patients with CID (Xie et al., 2020). Our findings support the “locus coeruleus neural fatigue” brain mechanism of the chronic insomnia disorder (Van Dort, 2016). Taken together, the decreased LC-NE functional network in prefrontal control network indicated the decreased cognitive control ability in patients with CID.

Previous studies have found gender difference in LC functional connectivity network in healthy control participants. For instance, Zhang et al. (2016) found that men show greater LC FC in the left hippocampus/parahippocampus and the left middle temporal gyrus than women. The results of the present study displayed that males show greater functional connectivity between LC and left amygdala and right MFG than women. As the amygdala and the MFG are important for the emotion and cognitive control process (McDougle et al., 1995; Szabadi, 2013), the gender difference might indicate different emotion and cognitive regulation strategies for insomnia. Further study might investigate the gender detail in the CID patients.

Interestingly, although posterior and frontal abnormalities in the LC-NE ascending pathway were found in patients in the CID group, such alterations were associated with different clinical manifestations. The posterior sensory cortex pathway (MOG) of the LC-NE system was associated with the duration of disease, while that of the frontal cognitive control pathway (dACC) was associated with anxiety symptoms. The prefrontal cortex is the main ascending projections in LC-NE system (Szabadi, 2013) involved in attentional and cognitive control. Recent retrograde tract-tracing experiments and optogenetic techniques also revealed target-specific projections in the LC-prefrontal cortex pathway (Chandler et al., 2014; Bari et al., 2020). The lower functional connectivity between LC and ACC have also found that in patients with late-life major depressive disorder, the LC-ACC FC was correlated with depressive severity (Del Cerro et al., 2020a). Our findings showing the LC-dACC pathway were associated with anxiety symptoms in CID patients, supporting the role of dorsal ACC in the anxiety in these patients. It should be recognized that the amygdala is the core region for emotional processing mediating fear and anxiety response in LC-NE system (McDougle et al., 1995; Szabadi, 2013); however, we did not find abnormal LC-amygdala FC in the CID group. Future studies need to verify this result. The occipital cortex is the main LC-visual cortex pathway projection involved in the modulation of visual sensory processing (McBurney-Lin et al., 2019), the increased fractional amplitude of low-frequency fluctuations (fALFF) in bilateral MOG was reported in participants with insomnia (Liu et al., 2016). Thus, the association of insomnia duration and LC-MOG pathway might indicate a mechanism that involves the hyperarousal state of the visual cortex in CID patients. Taken together, the clinical significance of the altered LC-NE system in CID patients suggests that different neural mechanisms underlie insomnia and emotional symptoms in CID and may help develop more targeted treatment strategies for CID patients with anxiety.

## Limitations

The present preliminary study has some limitations. First, the LC is also involved in another neurotransmitter system, which is beyond its primary definition as a NE-producing nucleus, e.g., the GABAergic circuit (Breton-Provencher and Sur, 2019). In addition, NE interacts with the serotonin and dopamine systems, which also make an important contribution to the mood regulation (Guiard et al., 2008). Thus, further study could explore the alterations in other neurotransmitter brain functional systems and their interaction in the insomnia. Second, we did not evaluate the cognition performance in the CID group; however, there is a large body of evidence which suggests that the LC-NE system is engaged in many cognitive processes, including attention and memory retrieval and consolidation, as well as decision making (Sara, 2009). Third, this study was a cross-sectional study; thus, the abnormal LC-NE-associated anxiety symptoms could not be explained as a causal relationship. Further longitudinal studies are required to verify our speculation. Lastly, as the anxiety disorder has also been associated with insomnia symptoms, further studies are required to reveal the potential LC-NE system relationship between insomnia symptoms in anxiety disorder population.

## Conclusion

Our study found increased FC in the LC-sensory cortex and decreased FC in the LC-prefrontal cortex in patients with CID. In addition, the altered LC-NE function in dACC was associated with anxiety symptoms in patients with CID. Our study provided evidence for abnormal LC-NE functional network in patients with CID. The present study substantially extended our understanding of the neuropathological basis of CID and provided a potential treatment target for CID patients with anxiety.

## Data Availability Statement

The original contributions presented in the study are included in the article/supplementary material, further inquiries can be directed to the corresponding author.

## Ethics Statement

The studies involving human participants were reviewed and approved by Institutional Review Board of The Third Affiliated Hospital of Anhui Medical University. The patients/participants provided their written informed consent to participate in this study.

## Author Contributions

LG and CX designed the experiments. LG and MS wrote the manuscript. JW, RX, XD, and SY conducted the statistical analysis. BZ, DL, and XZ contributed to the manuscript revision. MS and CX collected the data. All authors contributed to the article and approved the submitted version.

## Conflict of Interest

The authors declare that the research was conducted in the absence of any commercial or financial relationships that could be construed as a potential conflict of interest.

## Footnotes

1 http://www.fil.ion.ucl.ac.uk/spm

2 http://rfmri.org/dpabi

3 https://afni.nimh.nih.gov/pub/dist/doc/program_help/3dClustSim.html

4 https://www.alivelearn.net/xjview/
